# Mediterranean diet score is associated with greater allocentric processing in the EPAD LCS cohort: A comparative analysis by biogeographical region

**DOI:** 10.3389/fragi.2022.1012598

**Published:** 2022-12-09

**Authors:** Sarah Gregory, Craig W. Ritchie, Karen Ritchie, Oliver Shannon, Emma J. Stevenson, Graciela Muniz-Terrera

**Affiliations:** ^1^ Edinburgh Dementia Prevention, Centre for Clinical Brain Sciences, University of Edinburgh, Edinburgh, United Kingdom; ^2^ Institut National de la Santé et de la Recherche Médicale (INSERM), E361 Epidemiology of Nervous System Pathologies, La Colombière Hospital, Montpelier, France; ^3^ Human Nutrition Research Centre, Faculty of Medical Sciences, Population Health Sciences Institute, Newcastle University, Newcastle upon Tyne, United Kingdom; ^4^ Department of Social Medicine, Ohio University, Athens, OH, United States

**Keywords:** Alzheimer disease, Mediterranean diet, risk factor, cohort study, cognition

## Abstract

**Background:** Adherence to the Mediterranean diet (MedDiet), a primarily plant-based eating pattern, has been associated with lower dementia incidence. Much of the research has focused on Alzheimer’s disease (AD) dementia and mild cognitive impairment (MCI), with less research looking at the preclinical symptomatically silent stages that pre-empt MCI and AD dementia. Although there is evidence from studies conducted globally, no studies have compared the effects of the MedDiet within and outside of the Mediterranean region in one cohort.

**Methods:** Our study explored cross-sectional and longitudinal associations between MedDiet and cognition in the pan-European EPAD LCS, comparing those living within and outside of the Mediterranean region (as classified by European Union biogeographical definitions). After deriving MEDAS scores to quantify adherence to the MedDiet, we used linear regression and linear mixed effects models to test for associations between the MEDAS score and cognitive function measured by the Four Mountains Test (FMT) and the Repeatable Battery for the Assessment of Neuropsychological Status (RBANS). We additionally calculated MEDAS continuous and PYRAMID scores to provide alternative measures of MedDiet adherence.

**Results:** We included 1826 participants, mean age 65.69 (±7.42) years, majority female (56.2%) with family history (65.8%) and minority APOEε4 carriers (38.9%). Higher MEDAS scores were associated with better performance on the FMT both cross-sectionally (*n* = 1,144, ß: −0.11, SE: 0.04, *p* = 0.007) and longitudinally (slope: 0.10, 95% CI: 0.04–0.17, *p*: 0.002). The effect was marginally greater in the Mediterranean region in the cross-sectional analysis, with a stronger effect emerging longitudinally. In exploratory analyses, the association between MEDAS and FMT scores was only seen in female participants. A sensitivity analysis excluding Toulouse and Perugia, as cities near, but not within, the biogeographical region, found significant associations between higher MEDAS and MEDAS continuous scores, and a number of RBANS total and index scores.

**Conclusion:** MedDiet adherence is associated with better FMT scores, with effects seen most strongly in the Mediterranean region from longitudinal data. Our sensitivity analysis suggested a more global cognitive benefit of MedDiet adherence. This study highlights the need to further explore for whom and for what brain health outcomes the MedDiet confers benefit. This evidence would identify a window of opportunity in the life-course to maximise the benefit and better inform public health campaigns and patient-level interventions.

## 1 Introduction

The Mediterranean diet (MedDiet) is a primarily plant-based eating pattern, characterised by a high consumption of fruit, vegetables, legumes, nuts, olive oil and fish, a moderate consumption of red wine and a low consumption of red meat and processed foods (Trichopoulou et al., 2015). The MedDiet has been associated with multiple health benefits, including the maintenance of good brain health (SACN, 2018; Scarmeas et al., 2018). High adherence to a MedDiet has been associated with a 10%–40% lower incidence of dementia, particularly Alzheimer’s disease (Yusufov et al., 2017; Scarmeas et al., 2018). This association has primarily been shown in observational studies conducted within the Mediterranean basin.

Most studies investigating the effects of the MedDiet on brain health have focused on cognition. A systematic review exploring effects of MedDiet on cognition in RCTs reported non-significant or small effects sizes across the five included papers (Radd-Vagenas et al., 2018). Of the included studies one enrolled in the Mediterranean region and four outside of it. A second systematic review including observational studies also highlighted cognitive benefits of the MedDiet when enriched with pork, dairy or olive oil on processing speed and general cognitive function (Gutierrez et al., 2021). There are many proposed mechanisms explaining this apparent association between MedDiet adherence and cognition. As cited by this recent review (Siervo et al., 2021), these mechanisms include effects of the MedDiet on oxidative stress, systolic blood pressure, inflammation, and insulin resistance, all of which have implications for brain health.

The most consistent results from studies exploring MedDiet adherence and cognition are seen in Mediterranean countries. The PREDIMED trial (Spanish results reported here) found that participants randomised to MedDiet with supplemental extra virgin olive oil or nuts performed significantly better on a battery of cognitive tests compared to the control group in both the Navarra and Barcelona cohorts (Martínez-Lapiscina et al., 2013; Valls-Pedret et al., 2015). The InCHIANTI study (Italy) identified long term effects (average follow up period 10.1 years) of the MedDiet on yearly average MMSE decline, whereby those in the high and medium consumption groups experienced significant attenuation of decline (Tanaka et al., 2018). Meanwhile, the Three-City Study (France) found higher adherence to the MedDiet was associated with fewer MMSE errors over time (Féart et al., 2009), with intensive olive oil consumption, defined as used in cooking and dressing, associated with fewer deficits in visual memory and verbal fluency at baseline as well as less decline in visual memory performance over time (Berr et al., 2009).

Evidence for a beneficial effect of the MedDiet on cognition in non-Mediterranean countries is mixed. Higher MedDiet adherence as defined by the Pyramid score, was associated with better global cognitive function, verbal episodic memory, and processing speed performances in an aging UK cohort (Shannon et al., 2019). Adherence to the MedDiet, using a well-established scoring methodology (Panagiotakos et al., 2007), was also associated with a slower rate of cognitive decline in the Memory and Ageing project, a cohort based in the USA (Tangney et al., 2011). Analysis of a cohort study recruiting from the Hispanic community similarly found a significant association between MedDiet adherence, better cognitive performance at the first visit and a slower cognitive decline over 7 years compared to those with lower adherence (Moustafa et al., 2022). A randomized control trial that allocated participants in the USA with normal cognition and MCI to either the MedDiet or Western diet found a borderline significant interaction between diet, diagnosis and time for the cognitive composite, however no individual comparisons between the groups were significant for the cognitive outcome (Hoscheidt et al., 2021). However other observational studies in the UK using the MeDi score (Zhang et al., 2021) and USA using an adapted MeDi score analysing exclusively female participants (Samieri et al., 2013), as well as a clinical trial in Australia using an Australianised MedDiet score (Knight et al., 2016), found no significant benefits of MedDiet adherence across multiple cognitive domains. A future study from Australia analysing data from a cohort study also found no significant associations between the MedDiet, cognition or cognitive decline over 6 years (Chen et al., 2022). This apparent conflict of results between Mediterranean and non-Mediterranean countries, as well as the possibility of sex differences raised by the non-significant results from the UK Women’s Cohort Study and the Women’s Health Study, warrants further investigation.

The apparent differences between the effect of the MedDiet within and outside of the biogeographical Mediterranean region may be due to a multitude of factors. Firstly adherence to a MedDiet has been more common in Southern and European Mediterranean compared to non-Mediterranean countries for a number of years, and although adherence scores are decreasing in Mediterranean countries, they do, on average, remain higher than other countries (Vilarnau et al., 2019). Despite ongoing changes to the food supply common to European Mediterranean countries, resulting in increases in meat and dairy (Garcia-Closas et al., 2007), it may be that the core items remaining, such as olive oil, confer the most benefit to brain health (Khalatbary, 2013; Millman et al., 2021).

Our aim in this study was to test the hypothesis that adherence to a MedDiet would be associated with better cognitive function in a European cohort of participants living at risk for Alzheimer’s disease (AD) dementia. In addition, we aimed to perform a stratified analysis of those living within and outside of the Mediterranean region and an exploratory analysis stratified by sex.

## 2 Materials and methods

We used data from the European Prevention of Alzheimer’s Dementia Longitudinal Cohort Study (EPAD LCS) for this study. This cohort was selected as it recruited participants both within and outside of the Mediterranean region, allowing an exploration of differences between dietary associations with cognition by region. Explained in full elsewhere (Ritchie et al., 2016; Solomon et al., 2018), briefly the EPAD LCS is a pan-European cohort of participants recruited across a spectrum of risk states for AD dementia. Participants were eligible for inclusion in the EPAD LCS if they were aged 50 years or older, were in generally good health, were able to undergo an MRI and lumbar puncture, did not have dementia at baseline and had a study partner. Participants underwent a baseline visit with several assessments and were followed up at 6 months and then once a year for the duration of the project (2015–2020). Due to the ongoing recruitment throughout the project, participants have varying lengths of follow up (from one visit up to six visits). Participants consented for their data to be used in future research projects and data from the EPAD LCS is fully open access. After completing a data access request, following the EPAD procedures, we accessed the data from the Alzheimer’s Disease Drug Foundation (ADDF) platform. We used data from baseline, year 1, year 2 and year 3 for this study. The main focus of the study was to test for cross-sectional associations. Additional analyses were conducted to explore longitudinal cognitive performance and Mediterranean Diet Adherence Screener (MEDAS) scores.

### 2.1 Ethics and consent

Ethical approval was obtained in each country participating in the EPAD LCS, and any local governance procedures were followed at each site. All participants provided written informed consent before undergoing any study related activity. Informed consent was documented after the participant had the opportunity to read the approved information sheets and discuss the study with a trained and delegated member of the research team. All participants were required to have the capacity to consent at the time of study entry and dementia was an exclusion criterion at the baseline visit.

### 2.2 Data

We used the EPAD LCS v.IMI dataset for this analysis, following approval of a data access request (ep-ad.org/open-access-data/overview). EPAD LCS is registered at www.clinicaltrials.gov Identifier: NCT02804789. The dataset includes data from all participants who consented to join the study. Data used in preparation of this article were obtained from the EPAD LCS data set V.IMI, doi:10.34688/epadlcs_v.imi_20.10.30. We excluded participants with missing data in the exposure, outcome, and covariate variables of interest from the analysis. To determine minimum sample size we used G*Power Version 3.1. A sample size of 148 participants, with 74 participants in each group, was the minimum required for an *α* of 0.05 and a power of 0.95.

### 2.3 Sociodemographic data

Participants self-reported sociodemographic data through interviews with research staff. For this analysis we were interested in age (in years), education (in years), family history of dementia, sex and whether the site they were attending was based in the Mediterranean, which was used as a proxy for region of residence. We used the European Commission biogeographical region to classify a site as being Mediterranean or non-Mediterranean (European Commission). A list of site allocation is provided in the Supplementary Material (Supplementary Table S1). Both Toulouse and Perugia were situated near the border and for the main analysis are included in the Mediterranean region following consensus discussion by the analytical team. In addition, we conducted a sensitivity analysis with both sites excluded from the Mediterranean region analysis. The results are presented below and in the Supplementary Material.

### 2.4 Diet questionnaire and dietary score calculations

Participants were asked to complete a dietary questionnaire at baseline and year one reporting on their usual dietary habits for several MedDiet components. The questionnaire was developed for the HATICE study, designed to serve as a MedDiet adherence screener and is available on request (Richard et al., 2019). Participants reported their average consumption each day or week (depending on the question) for 46 items relevant to the MedDiet, including meats, fish and seafood, vegetables, fruits, nuts, legumes, olive oil and alcohol. Three MedDiet scores were calculated for each participant, the MEDAS score, the MEDAS continuous and the MedDiet Pyramid (Pyramid) score using previously published scoring methods. Full details of scoring methodologies are available in the Supplementary Material (Supplementary Table S2). The MEDAS score was used as the primary outcome measure as recommended by the authors of the adherence screener. Initially food responses were calculated as grams per day. Where required by the scoring methodology these scores were then converted to grams per week. From this portions per day or week where then calculated. Briefly the MEDAS score was calculated using a binary scoring method, whereby participants were allocated 0 or one points for each of 14 food groups depending on whether they met consumption criteria (Estruch et al., 2018). The MEDAS continuous was developed by Shannon et al (2019) with points allocated for same consumption criteria as MEDAS but on a continuous scale from 0 to 1, as opposed to binary allocations (Shannon et al., 2019). Similarly the Pyramid score is also coded continuously based on 15 points (Tong et al., 2016). Continuous scores have been shown to have more sensitivity to detecting differences in diet quality, particularly in a UK population (Shannon et al., 2019), and so were included given the large non-Mediterranean population enrolled in the EPAD LCS.

### 2.5 Cognition

Participants completed a battery of neuropsychological assessments at the baseline, year 1, year 2 and year 3, combining both validated and experimental tasks. Additionally, participants completed cognitive testing at month 6. However, as no dietary data is available at this visit this data was excluded. For this analysis we selected the Four Mountains Test (FMT) (Chan et al., 2016) and the Repeatable Battery for the Assessment of Neuropsychological Status (RBANS) (Karantzoulis et al., 2013). The FMT is a task assessing allocentric processing, with participants asked to initially study an image of four mountains and after a brief interval select which one of the four images then presented matches the stimulus but shown from a different point of view. We calculated total scores for each participant with higher scores indicating better performance. As the FMT is a novel research tool there are no normative scores. The RBANS is a clinically validated battery used both clinically and for research in mild cognitive impairment and AD dementia. Participants complete several cognitive tasks which are used to calculate cognitive domain indexes. For this study we used the RBANS total scale index as well as the individual index scores (attention, immediate memory, delayed memory, language, visuospatial constructional). We selected these two cognitive tests to understand differences in both early and more progressed global cognitive impairment. The FMT has been developed by cognitive sciences to specifically target the brain regions first affected by early tau and amyloid accumulation, namely the hippocampus, entorhinal cortex, precuneus and retrosplenial cortex (Nestor et al., 2003; Rowe et al., 2007; Chen et al., 2010; Khan et al., 2014; Ritchie et al., 2017). Conversely performance on the RBANS differences between those with and without greater global deterioration across a number of cognitive domains (Ritchie et al., 2017).

### 2.6 APOE

At baseline all participants provided a DNA sample which was used for *APOEe4* genotyping. This analysis was carried using Taqman Genotyping in a single laboratory on QuantStudio12K Flex, with further details available in the EPAD LCS v500.0 baseline paper (Ritchie et al., 2020). Participants were classified as carriers (regardless of whether they were heterozygous or homozygous for *APOEe4* allele) or non-carriers.

### 2.7 Physical health and medical history

Participants self-reported their smoking history as current, former or non-smokers. Medical histories were collected, and the following variables were selected as covariates in this analysis, selected due to their theoretical association with diet: hypertension, hypercholesterolemia, hyperglycaemia, diabetes, stroke, antihypertensive medication use and diabetic medication use. All measures were binary, categorised as presence or absence of diagnosis or medication use. Height and weight were measured during the participant’s site visit and were used to calculate the body mass index (BMI) which was included as a continuous variable. Participants completed the Pittsburgh Sleep Quality Index (PSQI) questionnaire (Buysse et al., 1989) with higher total scores indicating poorer sleep quality. The scores were used as a continuous measure in the regression models. Self-reported physical activity levels (ranging from never to daily) were extracted and included as a covariate.

### 2.8 Statistical analysis

All statistical analyses were done in R (Version 4.1.0). Participants with missing data in the variables of interest were excluded from the analysis. Descriptive statistics were calculated for all participants. We initially tested the cohort as a whole and fitted univariate and fully adjusted linear regression models to test for associations between MEDAS and the co-primary outcomes (FMT, RBANS) at baseline. The fully adjusted model included the following variables: sex, age, education, family history, *APOE,* physical activity, smoking, sleep, BMI, hypertension, hypercholesterolemia, hyperglycaemia, diabetes, stroke, antihypertensive medication use, diabetic medication use, living in the Mediterranean. All covariates were included in the models simultaneously. We tested for interactions between dietary score and region lived in. Our pre-planned stratified analysis split the dataset into participants living in Mediterranean and non-Mediterranean regions and the previously mentioned analysis was replicated with living in the Mediterranean removed as a covariate from the fully adjusted models. We then repeated the analysis with the MEDAS continuous and then the Pyramid scores to explore whether different definitions of MedDiet adherence impacted the observed associations. Finally, we ran our exploratory analysis repeating the above with the cohort stratified by sex, initially as a whole cohort and then by Mediterranean region. We also analysed the longitudinal associations between diet and cognition across all visits, using linear mixed effect models. We modelled for random effects of participant and visit, and included the same covariates as in previous models, initially running the models in the whole cohort and then splitting by biogeographical region.

## 3 Results

We included 1826 participants in the RBANS analysis and 1,144 participants in the FMT analysis. Compared to participants who were excluded due to missing data, the participants included in either analysis were more likely to be *APOE*e4 carriers (RBANS: 38.9% vs. 21.6%, *p* < 0.001; FMT: 39.2% vs. 33.6%, *p* < 0.001), were younger in the RBANS, but not the FMT cohort (65.69 ± 7.42 years vs. 67.27 ± 8.00 years, *p*: 0.001) and were more likely to live in the Mediterranean region in either analysis (RBANS: 33.24 vs. 23.8%, *p* = 0.002; FMT: 38.2% vs. 24.8%, *p* < 0.001). It may be that the participants with full data were more motivated or able to complete the assessments, however this is just speculative. Full descriptive details of the cohort are presented in Table 1.

**TABLE 1:** Descriptive statistics of participants included in the RBANS and Four Mountains Test, FMT analysis.

Variable (n (%) unless otherwise stated)	RBANS included (*n* = 1826)	FMT included (*n* = 1,144)
APOE Carrier	710 (38.9)	448 (39.2)
Non-carrier	1116 (61.1)	696 (60.8)
Family history of dementia	1202 (65.8)	776 (67.8)
No family history	624 (34.2)	368 (32.2)
Female	1027 (56.2)	658 (57.5)
Male	799 (43.8)	486 (42.5)
Age in years [mean (SD)]	65.69 (7.42)	66.18 (7.04)
Years of education [mean (SD)]	14.38 (3.73)	14.61 (3.67)
Mediterranean region	609 (33.4)	437 (38.2)
MEDAS score [mean (SD)]	7.26 (1.68)	7.14 (1.71)
MEDAS continuous [mean (SD)]	8.46 (1.52)	8.36 (1.55)
Pyramid score [mean (SD)]	6.99 (1.42)	6.91 (1.43)
Total cognitive test score [mean (SD)]	101.96 (15.35)	9.24 (2.56)

### 3.1 Associations between dietary scores and cognitive tests at baseline

Higher MEDAS scores were associated with better performance on the Four Mountains Test in unadjusted and fully adjusted models (Unadjusted model β: 0.16, SE: 0.04, *p*: 0.0003; Fully adjusted model β: 0.11, SE: 0.04, *p*: 0.007). Higher Pyramid scores were associated with lower RBANS attention index scores in the fully adjusted (but not unadjusted) model (β: −0.58, SE: 0.27, *p*: 0.04). There were no significant associations seen with the MEDAS continuous or Pyramid scores with the FMT (although a similar, non-significant pattern seen with higher MEDAS continuous scores and higher FMT scores), and no significant associations between any MedDiet score and either total or any other individual index of the RBANS. These results suggest that higher adherence to the MedDiet is associated with better performance on the FMT (better brain health) and may be associated with poorer attention based on the RBANS attention index score although this relationship is less clear as this was only significant in the fully and not unadjusted model. Full details of all models are presented in Table 2. There was a significant interaction of Pyramid score and living outside the Mediterranean region on RBANS immediate memory score, whereby higher Pyramid scores were associated with better performance on this task within this region (fully adjusted model interaction: β: 1.41, SE: 0.53, *p*: 0.008). There were no other significant interactions between dietary scores and region on any of the other cognitive outcome measures.

**TABLE 2:** Fully adjusted linear regression models of MedDiet scores (MEDAS, MEDAS Continuous and Pyramid) with cognitive outcomes, MedDiet score, sex, age, education and APOE shown. Fully Adjusted Model: MedDiet Score, sex, age, education, family history, APOE, physical activity, smoking, sleep, body mass index, hypertension, hypercholesterolemia, hyperglycaemia, diabetes, stroke, antihypertensive medication use, diabetic medication use. AI, attention index; DMI, delayed memory index; FMT, Four Mountains Test; IMI, immediate memory index; LI, language index; m, male; RBANS, Repeatable Battery for the Assessment of Neuropsychological Status; VCI, visuoconstructional index.

	FMT			RBANS total			AI			DMI			IMI			LI			VCI		
MEDAS																					
	β	SE	*p*	β	SE	*p*	β	SE	*p*	β	SE	*p*	β	SE	*p*	β	SE	*p*	β	SE	*p*
Dietary score	0.12	0.04	0.004	−0.18	0.20	0.37	−0.23	0.23	0.32	−0.05	0.23	0.82	0.13	0.21	0.54	−0.19	0.16	0.22	−0.24	0.22	0.29
Age	−0.13	0.01	<0.001	−0.24	0.05	<0.001	−0.23	0.06	<0.001	−0.26	0.05	<0.001	−0.29	0.05	<0.001	−0.05	0.04	0.15	−0.07	0.05	0.18
Sex (m)	0.71	0.15	<0.001	−2.58	0.69	<0.001	−0.02	0.81	0.98	−3.00	0.79	<0.001	−.38	0.72	<0.001	−5.41	0.55	<0.001	3.80	0.77	<0.001
Education	0.08	0.02	<0.001	1.28	0.09	<0.001	1.19	0.11	<0.001	0.89	0.10	<0.001	0.97	0.09	<0.001	0.50	0.07	<0.001	1.03	0.10	<0.001
APOE (no)	0.19	0.14	0.18	1.45	0.69	0.03	0.09	0.80	0.91	2.86	0.78	<0.001	3.38	0.71	<0.001	−0.04	0.54	0.95	−0.74	0.76	0.33
MEDAS continuous																					
Dietary score	0.07	0.05	0.12	−0.25	0.22	0.27	−0.37	0.26	0.15	−0.08	0.25	0.75	0.19	0.23	0.41	−0.13	0.17	0.45	−0.46	0.25	0.06
Age	−0.13	0.01	<0.001	−0.24	0.05	<0.001	−0.23	0.06	<0.001	−0.26	0.05	<0.001	−0.29	0.05	<0.001	−0.05	0.04	0.17	−0.07	0.05	0.19
Sex (m)	0.69	0.15	<0.001	−2.57	0.69	<0.001	−0.01	0.81	0.99	−3.00	0.78	<0.001	−4.38	0.72	<0.001	−5.39	0.54	<0.001	3.80	0.77	<0.001
Education	0.08	0.02	<0.001	1.27	0.09	<0.001	1.18	011	<0.001	0.89	0.10	<0.001	0.97	0.09	<0.001	0.49	0.07	<0.001	1.02	0.10	<0.001
APOE (no)	0.19	0.14	0.19	1.48	0.69	0.03	0.15	0.80	0.86	2.88	0.78	<0.001	3.36	0.71	<0.001	−0.04	0.54	0.95	−0.66	0.76	0.39
Pyramid																					
Dietary score	0.009	0.05	0.85	−0.18	0.23	0.45	−0.58	0.27	0.04	0.20	0.27	0.46	0.33	0.24	0.18	−0.14	0.18	0.45	−0.47	0.26	0.07
Age	−0.14	0.01	<0.001	−0.24	0.69	<0.001	−0.22	0.06	<0.001	−0.26	0.05	<0.001	−0.29	0.05	<0.001	−0.05	0.04	0.18	−0.07	0.05	0.22
Sex (m)	0.68	0.15	<0.001	−2.54	0.05	<0.001	0.04	0.81	0.96	−3.00	0.78	<0.001	−4.41	0.72	<0.001	−5.38	0.54	<0.001	3.85	0.77	<0.001
Education	0.08	0.02	<0.001	1.27	0.09	<0.001	1.18	0.11	<0.001	0.89	0.10	<0.001	0.97	0.09	<0.001	0.49	0.07	<0.001	1.02	0.10	<0.001
APOE (no)	0.20	0.14	0.16	1.45	0.69	0.03	0.14	0.80	0.86	2.83	0.78	<0.001	3.35	0.71	<0.001	−0.05	0.54	0.93	−0.71	0.76	0.36

### 3.2 Stratification by biogeographical region

Our pre-planned secondary analysis was to explore differences between associations of the MedDiet and cognitive function by residential region (Mediterranean vs. non-Mediterranean). Participants living in the Mediterranean [RBANS *n* = 609 (33.4%); FMT *n* = 437 (38.2%)] were more likely to be APOEe4 carriers, were less likely to have a family history of dementia, had less education and in the FMT group were younger (RBANS *n* = 1,217; FMT *n* = 707). Participants living in the Mediterranean region had significantly higher MEDAS, MEDAS continuous and Pyramid scores compared to participants living in non-Mediterranean regions. Full details on demographics in the two regions are presented in Table 3.

**TABLE 3:** Comparison of descriptive statistics between participants living in Mediterranean and non-Mediterranean countries *p < 0.05; ***p < 0.001.

Variable (n (%) unless otherwise stated)	RBANS			FMT		
	Mediterranean (*n* = 609)	Non-mediterranean (*n* = 1,217)	*p* test	Mediterranean (*n* = 437)	Non-mediterranean (*n* = 707)	*p* test
APOE Carrier	207 (34)	503 (41.3)	0.003**	135 (30.9)	313 (44.3)	<0.001***
Non-carrier	402 (66)	714 (58.7)	302 (69.1)	394 (55.7)		
Family history	466 (76.5)	736 (60.5)	<0.001***	336 (76.9)	440 (62.2)	<0.001***
No family history	143 (23.5)	481 (39.5)		101 (23.1)	267 (37.8)	
Female	350 (57.5)	677 (55.6)	0.49	254 (58.1)	404 (57.1)	0.79
Male	259 (42.5)	540 (44.4)		183 (41.9)	303 (42.9)	
Age in years [mean (SD)]	65.93 (7.20)	65.58 (7.53)	0.34	65.57 (6.99)	66.56 (7.04)	0.02*
Years of education [mean (SD)]	13.51 (3.69)	14.82 (3.67)	<0.001***	13.74 (3.62)	15.16 (3.59)	<0.001***
MEDAS score [mean (SD)]	7.41 (1.63)	7.19 (1.70)	0.007**	7.37 (1.66)	7.00 (1.72)	<0.001***
MEDAS continuous [mean (SD)]	8.74 (1.40)	8.33 (1.56)	<0.001***	8.77 (1.41)	8.10 (1.58)	<0.001***
Pyramid score [mean (SD)]	7.15 (1.34)	6.91 (1.45)	<0.001***	7.15 (1.37)	6.77 (1.46)	<0.001***
Total cognitive test score	97.93 (15.99)	103.97 (14.62)	<0.001***	9.12 (2.56)	9.31 (2.56)	0.23

The association between higher MedDiet adherence, as measured by the MEDAS score, and higher FMT scores remained marginally significant in both the Mediterranean region (Unadjusted model β: 0.21, SE: 0.07, *p*: 0.005; Fully adjusted model β: 0.14, SE: 0.07, *p*: 0.049) and the non-Mediterranean region in the fully adjusted model (Unadjusted model β: 0.14, SE: 0.06, *p*: 0.01; Fully adjusted model β: 0.11, SE: 0.05, *p*: 0.45). Interestingly in the non-Mediterranean region higher MEDAS continuous scores were associated with better performance on the FMT (β: 0.13, SE: 0.06, *p*: 0.03), although this effect was attenuated in the fully adjusted model. For non-Mediterranean participants higher Pyramid score was associated with better performance on the RBANS Immediate Memory index in both unadjusted and fully adjusted models (Unadjusted model: β: 1.02, SE: 0.30, *p*: 0.0006; Fully adjusted model: β: 0.83, SE: 0.28, *p*: 0.003). Both the MEDAS and MEDAS continuous scores were also associated with this index in non-Mediterranean participants however these effects were attenuated in fully adjusted models. There were no other significant associations between any of the MedDiet scores and other RBANS total or index scores for participants in either region (Table 4).

**TABLE 4:** Linear regression models of MedDiet scores (MEDAS, MEDAS Continuous and Pyramid) with cognitive outcomes living in Mediterranean (FMT: *n* = 437; RBANS: *n* = 609) and non-Mediterranean countries (FMT: *n* = 707; RBANS: *n* = 1,217). Unadjusted Model: MedDiet Score; Fully Adjusted Model: MedDiet Score, sex, age, education, family history, APOE, physical activity, smoking, sleep, BMI, hypertension, hypercholesterolemia, hyperglycaemia, diabetes, stroke, antihypertensive medication use, diabetic medication use. *p < 0.05; **p < 0.01; ***p < 0.001.

Cognitive test	Mediterranean						Non-mediterranean					
	MEDAS		MEDAS continuous		Pyramid		MEDAS		MEDAS continuous		Pyramid	
	Unadjusted	Fully adjusted	Unadjusted	Fully adjusted	Unadjusted	Fully adjusted	Unadjusted	Fully adjusted	Unadjusted	Fully adjusted	Unadjusted	Fully adjusted
4 Mountains test	β: 0.21 SE: 0.07 p: 0.005**	β: 0.14 SE: 0.07 p: 0.049*	β: 0.08 SE: 0.09 p: 0.37	β: −0.02 SE: 0.08 p: 0.81	β: −0.006 SE: 0.09 p: 0.95	β: −0.06 SE: 0.08 p: 0.51	β: 0.14 SE: 0.06 p: 0.01*	β: 0.11 SE: 0.05 p: 0.045*	β: 0.13 SE: 0.06 p: 0.03*	β: 0.11 SE: 0.06 p: 0.07	β: 0.06 SE: 0.07 p: 0.37	β: 0.04 SE: 0.06 p: 0.48
RBANS total scale	β: 0.06 SE: 0.40 p: 0.89	β: −0.20 SE: 0.37 p: 0.59	β: 0.09 SE: 0.46 p: 0.85	β: −0.07 SE: 0.43 p: 0.87	β: −0.30 SE: 0.48 p: 0.54	β: −0.57 SE: 0.45 p: 0.21	β: 0.33 SE: 0.25 p: 0.18	β: −0.05 SE: 0.24 p: 0.85	β: 0.24 SE: 0.27 p: 0.38	β: -0.09 SE: 0.26 p: 0.74	β: 0.39 SE: 0.29 p: 0.18	β: 0.22 SE: 0.28 p: 0.42
RBANS attention index	β: 0.46 SE: 0.43 p: 0.29	β: 0.07 SE: 0.42 p: 0.87	β: 0.39 SE: 0.50 p: 0.44	β: 0.09 SE: 0.48 p: 0.86	β: −0.24 SE: 0.53 p: 0.65	β: −0.66 SE: 0.50 p: 0.19	β: 0.04 SE: 0.28 p: 0.89	β: −0.24 SE: 0.28 p: 0.39	β: −0.007 SE: 0.31 p: 0.98	β: −0.26 SE: 0.31 p: 0.41	β: -0.16 SE: 0.33 p: 0.64	β: −0.32 SE: 0.33 p: 0.434
RBANS delayed memory index	β: 0.16 SE: 0.46 p: 0.73	β: −0.05 SE: 0.44 p: 0.91	β: 0.29 SE: 0.53 p: 0.56	β: 0.15 SE: 0.51 p: 0.76	β: 0.06 SE: 0.56 p: 0.91	β: −0.05 SE: 0.53 p: 0.93	β: 0.35 SE: 0.27 p: 0.20	β: 0.03 SE: 0.26 p: 0.90	β: 0.20 SE: 0.29 p: 0.50	β: 0.003 SE: 0.29 p: 0.99	β: 0.66 SE: 0.31 p: 0.03*	β: 0.50 SE: 0.30 p: 0.10
RBANS immediate memory index	β: 0.08 SE: 0.42 p: 0.85	β: −0.17 SE: 0.40 p: 0.67	β: −0.06 SE: 0.48 p: 0.90	β: −0.20 SE: 0.46 p: 0.66	β: −0.44 SE: 0.51 p: 0.39	β: −0.65 SE: 0.48 p: 0.18	β: 0.75 SE: 0.25 p: 0.003**	β: 0.31 SE: 0.24 p: 0.21	β: 0.82 SE: 0.28 p: 0.003**	β: 0.43 SE: 0.27 p: 0.11	β: 1.02 SE: 0.30 p: 0.0006***	β: 0.83 SE: 0.28 p: 0.003**
RBANS language index	β: −0.08 SE: 0.26 p: 0.77	β: −0.04 SE: 0.26 p: 0.86	β: 0.24 SE: 0.30 p: 0.43	β: 0.37 SE: 0.30 p: 0.21	β: −0.30 SE: 0.32 p: 0.34	β: −0.27 SE: 0.31 p: 0.39	β: 0.03 SE: 0.20 p: 0.90	β: −0.16 SE: 0.20 p: 0.43	β: −0.01 SE: 0.22 p: 0.95	β: −0.17 SE: 0.22 p: 0.43	β: 0.12 SE: 0.24 p: 0.60	β: 0.10 SE: 0.23 p: 0.67
RBANS Visuo-constructional Index	β: −0.07 SE: 0.44 p: 0.88	β: −0.22 SE: 0.43 p: 0.61	β: −0.08 SE: 0.51 p: 0.88	β: −0.25 SE: 0.50 p: 0.62	β: −0.11 SE: 0.53 p: 0.84	β: −0.38 SE: 0.52 p: 0.47	β: 0.0001 SE: 0.03 p: 0.997	β: −0.12 SE: 0.26 p: 0.65	β: −0.25 SE: 0.29 p: 0.40	β: −0.41 SE: 0.29 p: 0.16	β: −0.28 SE: 0.31 p: 0.38	β: −0.34 SE: 0.30 p: 0.26

### 3.3 Sex-stratified analyses

There were no diet:sex interactions seen for any dietary score or cognitive outcome. Our exploratory sex-stratified analysis found that the association between higher MEDAS scores and better FMT performance was seen in female, but not male participants (Female, fully adjusted: β: 0.20, SE: 0.06, *p*: 0.0004; Male, fully adjusted: β: 0.03, SE: 0.06, *p*: 0.58). There were no other significant associations in fully adjusted models in either group, with full results available in Table 5. When we additionally separated by region, we see that this effect remains in the female participants living in non-Mediterranean regions (Unadjusted model: β: 0.22, SE: 0.07, *p*: 0.003; Fully adjusted model: β: 0.22, SE: 0.07, *p*: 0.003). There were no other significant associations seen in this sex- and region-stratified exploratory analysis (see Supplementary Table S3 for full details).

**TABLE 5:** Linear regression models of MedDiet scores (MEDAS, MEDAS Continuous and Pyramid) with cognitive outcomes living in male and female participants. Unadjusted Model: MedDiet Score; Fully Adjusted Model: MedDiet Score, age, education, family history, APOE, physical activity, smoking, sleep, BMI, hypertension, hypercholesterolemia, hyperglycaemia, diabetes, stroke, antihypertensive medication use, diabetic medication use, Mediterranean region. *p < 0.05; **p < 0.01; ***p < 0.001.

Cognitive test	Male						Female					
	MEDAS		MEDAS continuous		Pyramid		MEDAS		MEDAS continuous		Pyramid	
	Unadjusted	Fully adjusted	Unadjusted	Fully adjusted	Unadjusted	Fully adjusted	Unadjusted	Fully adjusted	Unadjusted	Fully adjusted	Unadjusted	Fully adjusted
4 Mountains test	β: 0.13 SE: 0.07 p: 0.05	β: 0.02 SE: 0.06 p: 0.70	β: 0.13 SE: 0.07 p: 0.07	β: 0.009 SE: 0.07 p: 0.89	β: 0.02 SE: 0.08 p: 0.77	β: −0.04 SE: 0.08 p: 0.59	β: 0.20 SE: 0.06 p: 0.0008***	β: 0.21 SE: 0.06 p: 0.0002***	β: 0.07 SE: 0.07 p: 0.25	β: 0.09 SE: 0.06 p: 0.13	β: 0.03 SE: 0.07 p: 0.69	β: 0.01 SE: 0.06 p: 0.83
RBANS total scale	β: −0.08 SE: 0.31 p: 0.79	β: −0.36 SE: 0.30 p: 0.22	β: 0.02 SE: 0.35 p: 0.96	β: −0.23 SE: 0.33 p: 0.49	β: −0.20 SE: 0.38 p: 0.59	β: −0.38 SE: 0.36 p: 0.29	β: 0.26 SE: 0.29 p: 0.37	β: −0.09 SE: 0.27 p: 0.75	β: −0.15 SE: 0.32 p: 0.64	β: −0.34 SE: 0.30 p: 0.26	β: 0.20 SE: 0.34 p: 0.55	β: -0.12 SE: 0.31 p: 0.71
RBANS attention index	β: 0.11 SE: 0.36 p: 0.76	β: −0.18 SE: 0.36 p: 0.61	β: 0.06 SE: 0.40 p: 0.88	β: −0.22 SE: 0.40 p: 0.58	β: −0.27 SE: 0.44 p: 0.53	β: −0.38 SE: 0.09 p: 0.37	β: −0.02 SE: 0.33 p: 0.95	β: −0.30 SE: 0.31 p: 0.34	β: −0.40 SE: 0.36 p: 0.26	β: −0.49 SE: 0.35 p: 0.16	β: −0.49 SE: 0.38 p: 0.20	β: −0.72 SE: 0.37 p: 0.047
RBANS delayed memory index	β: −0.05 SE: 0.36 p: 0.89	β: −0.32 SE: 0.35 p: 0.36	β: −0.04 SE: 0.40 p: 0.91	β: −0.32 SE: 0.39 p: 0.42	β: 0.10 SE: 0.43 p: 0.82	β: −0.18 SE: 0.42 p: 0.66	β: 0.37 SE: 0.31 p: 0.24	β: 0.14 SE: 0.30 p: 0.64	β: 0.09 SE: 0.34 p: 0.78	β: 0.02 SE: 0.33 p: 0.94	β: 0.61 SE: 0.36 p: 0.09	β: 0.38 SE: 0.35 p: 0.27
RBANS immediate memory index	β: 0.32 SE: 0.33 p: 0.34	β: -0.05 SE: 0.32 p: 0.88	β: 0.51 SE: 0.37 p: 0.17	β: 0.19 SE: 0.36 p: 0.59	β: 0.29 SE: 0.41 p: 0.48	β: -0.02 SE: 0.39 p: 0.95	β: 0.51 SE: 0.28 p: 0.07	β: 0.20 SE: 0.27 p: 0.46	β: 0.29 SE: 0.31 p: 0.35	β: 0.10 SE: 0.30 p: 0.73	β: 0.70 SE: 0.32 p: 0.03*	β: 0.46 SE: 0.32 p: 0.15
RBANS language index	β: −0.28 SE: 0.21 p: 0.19	β: −0.29 SE: 0.22 p: 0.18	β: −0.02 SE: 0.24 p: 0.94	β: −0.05 SE: 0.24 p: 0.84	β: −0.17 SE: 0.26 p: 0.52	β: −0.22 SE: 0.26 p: 0.39	β: −0.01 SE: 0.23 p: 0.95	β: −0.15 SE: 0.22 p: 0.51	β: −0.22 SE: 0.25 p: 0.38	β: −0.28 SE: 0.25 p: 0.26	β: 0.01 SE: 0.26 p: 0.97	β: −0.15 SE: 0.26 p: 0.56
RBANS Visuo-constructional index	β: −0.20 SE: 0.34 p: 0.57	β: −0.33 SE: 0.34 p: 0.34	β: −0.40 SE: 0.38 p: 0.30	β: −0.44 SE: 0.38 p: 0.25	β: −0.73 SE: 0.42 p: 0.08	β: −0.67 SE: 0.41 p: 0.10	β: 0.09 SE: 0.31 p: 0.77	β: −0.21 SE: 0.30 p: 0.48	β: −0.25 SE: 0.34 p: 0.47	β: −0.50 SE: 0.33 p: 0.13	β: −0.08 SE: 0.36 p: 0.81	β: −0.32 SE: 0.34 p: 0.35

### 3.4 Sensitivity analysis

We conducted a sensitivity analysis with the MedDiet scores to remove Toulouse and Perugia from the Mediterranean region as they sit on or close to the border of the bio-geographically defined region. Interestingly we see a significant effect of excluding these two sites on the MEDAS and MEDAS continuous scores (Supplementary Table S4). When Toulouse and Perugia are excluded, the significant positive association between higher MEDAS scores and higher FMT performance remains. In addition, there is a positive association between higher MedDiet scores, as measured by the MEDAS and MEDAS continuous on the RBANS total scale, the attention index, the delayed memory index (MEDAS continuous only), the language index and the visuo-constructional index (MEDAS only). There remain no significant associations between the Pyramid score and any of the cognitive tests, and no association with the immediate memory index and any of the MedDiet scores. These results should be interpreted in the context of a sensitivity analysis with reduced sample sizes (FMT: *n* = 262; RBANS: *n* = 393), however they suggest an influence of the Toulouse and Perugia sites on the overall results. This may indicate that the MedDiet is particularly impactful in the true Mediterranean region, and less so in borderline areas where dietary practices may blend those of Mediterranean and non-Mediterranean regions.

We conducted a further sensitivity analysis to remove those with a clinical dementia rating (CDR) score of 0.5, which is indicative of MCI (*n* = 262 in FMT dataset, *n* = 493 in RBANS dataset). This has no effect on the analysis when the data was considered as a whole cohort on any of the outcome measures. When splitting the data by region there was no longer a significant association between MEDAS score and FMT score for those in the Mediterranean region if people with MCI were excluded (fully adjusted β: 0.10, SE: 0.09, *p*: 0.24), however the association remained outside of the Mediterranean region (fully adjusted β: 0.13, SE: 0.06, *p*: 0.02). There were no associations seen in either region with any of the RBANS indexes when those with MCI were excluded from the dataset.

### 3.5 Longitudinal associations between diet and cognition

There were no significant longitudinal changes in diet as measured using the MEDAS score (Supplementary Table S5).

Linear mixed method models showed a significant association between the MEDAS scores and the FMT, whereby higher adherence to the MedDiet was associated longitudinally with higher FMT scores (n = 1,070; slope: 0.16, SE: 0.03, *t*: 4.54, 95% CI: 0.09, 0.22, *p* < 0.001; Adjusted: slope: 0.10, SE: 0.03, *t*: 3.13, 95% CI: 0.04, 0.17, *p*: 0.002). This association was seen in the Mediterranean region in both unadjusted and fully adjusted models (Unadjusted: slope: 0.20, SE: 0.05, *t*: 3.86, 95% CI: 0.10, 0.30, *p*: 0.0001; Fully adjusted: slope: 0.13, SE: 0.05, *t*: 2.55, 95% CI: 0.03, 0.22, *p*: 0.01), while only the unadjusted models were significant in the non-Mediterranean region (Unadjusted: slope: 0.12, SE: 0.05, *t*: 2.82, 95% CI: 0.04, 0.22, *p*: 0.005; Adjusted: slope: 0.08, SE: 0.04, *t*: 1.82, 95% CI: −0.005).

There were no significant longitudinal associations between the MEDAS score and the RBANS total score in unadjusted or fully adjusted models (*n* = 1825, slope: 0.07, SE: 0.12, *t*: 0.59, 95% CI: −0.16, 0.30, *p*: 0.56; Adjusted: slope: −0.03, SE: 0.11, *t*: −0.30, 95% CI: −0.26, 0.19, *p*: 0.76). This remained true when the data was split by biogeographical region (Mediterranean region: slope: −0.14, SE: 0.20, *t*: −0.72, 95% CI: −0.52, 0.24, *p*: 0.47; Non-Mediterranean region: slope: 0.25, SE: 0.14, t: 1.71, 95% CI: −0.04, 0.53, *p*: 0.09).

## 4 Discussion

This study found a greater adherence to the MedDiet, as measured by the MEDAS score, was associated with better performance on the FMT, both cross-sectionally and longitudinally. Participants living in the Mediterranean region had statistically significantly higher MedDiet scores across all three scoring methods, suggesting a higher adherence to the MedDiet compared to those living outside of the Mediterranean region. Our stratified analyses found no differences by biogeographical region at baseline, but a longitudinal association only for those living in the Mediterranean region. Interestingly we see the significant positive association between diet and FMT score in women but not men. Our sensitivity analysis, removing two sites that sat just outside of the border of where the EU commission classify the biogeographical region of the Mediterranean region showed a much broader positive effect of MedDiet adherence on cognition as measured by the RBANS total and index scores.

Our main finding that higher MEDAS score are associated with higher FMT scores, confirms previous research that has associated greater MedDiet adherence with better cognitive performance, although, as with previous research, effect sizes remain small (Radd-Vagenas et al., 2018; Gutierrez et al., 2021). Previous studies have found evidence of an effect of the MedDiet across a broad domain of cognition; however, our analysis only found a significant association with the FMT, a test of allocentric processing, with no significant associations seen in any of the RBANS total or index scores. The FMT is designed to be a test sensitive to the earliest changes associated with AD (Moodley et al., 2015; Ritchie et al., 2018), and in this pre-clinical population may be more appropriate than the RBANS. While many observational studies have conducted cross-sectional analyses, there is growing evidence that there are also longitudinal associations between diet and cognition, as we found in this study (Hossain et al., 2020; Coelho-Júnior et al., 2021; Wade et al., 2021). These findings, alongside the previous evidence base, suggest long term cognitive benefits of adhering to a MedDiet, particularly for those living within the Mediterranean. Considering only those who were cognitively healthy this association was no longer seen in the Mediterranean region but was still seen in the non-Mediterranean region. It may be that the MedDiet is beneficial for cognitive performance in those with disease living in the Mediterranean region, and beneficial for cognitive performance for cognitively healthy participants outside of the Mediterranean region. These findings may also reflect the fact that the sample size was significantly reduced by excluding those with MCI and splitting by region, or could reflect potential recall bias from those with MCI completing the dietary questionnaires and over or under reporting on dietary information.

One of our aims was to explore differences between those living within and outside of the Mediterranean region, to understand if the diet conferred more benefit within the region. Stratified analysis showed a small effect of the diet in the Mediterranean region on FMT performance, with a borderline significant effect in the non-Mediterranean region. Considering our sensitivity analysis, which removed Toulouse and Perugia from the Mediterranean, we see significant positive associations between higher diet adherence (measured by either the MEDAS or MEDAS continuous scores) and cognition across a multitude of measures, including the FMT. These scores can only be treated as exploratory and do involve a much smaller sample size compared to the main analysis. However, the results suggest that the MedDiet may confer greater benefits to those living within the Mediterranean region. Previous studies found associations in Mediterranean countries, and although some sites would be in the over the biogeographical border, most do sit within the true Mediterranean region (Berr et al., 2009; Féart et al., 2009; Martínez-Lapiscina et al., 2013; Valls-Pedret et al., 2015; Tanaka et al., 2018). One hypothesis to explain this possible difference in effect by biogeographical region is life-long adoption of the diet, compared to more recent dietary adaptation, along with ease of access to the food components in a MedDiet. Although there has been an increase in the global access to MedDiet components since the 1960s, some key foods such an olive oil remain much more available within the Mediterranean region (Vareiro et al., 2009). The transferability of the MedDiet outside the Mediterranean is certainly possible, however does bear challenges such as challenging common misconceptions about food components of the diet and often ignoring the need to restrict or avoid certain food groups (Martínez-González et al., 2017). There may also be unmeasured effects on the style of eating, with a convivial social approach to meals commonplace in the Mediterranean (de la Torre-Moral et al., 2021).

Our final analysis investigated the differential association of MedDiet with cognitive outcomes by sex. Contrary to published research we found a significant association between the MEDAS score and FMT performance in female participants rather than male participants. Interestingly in further exploratory work this was only seen in participants living outside of the Mediterranean region, despite previous studies in the UK and USA finding no benefit of the MedDiet on cognition for female participants (Samieri et al., 2013; Zhang et al., 2021). The analysis of the UK Women’s Cohort study tested for associations between MedDiet and reaction time a cohort of women in their 50s (Zhang et al., 2021). Reaction time may be a much less sensitive test of cognitive function compared to the FMT included in our task, and the cohort was over 10 years younger on average than the EPAD LCS, both of which may explain the difference in results between our studies. Analysis of the Women’s Health Study used a comprehensive cognitive battery recruiting a similar age group to our analysis (Samieri et al., 2013). The results we see in the RBANS may reflect the results from the Women’s Health Study, with the FMT being a more sensitive test than either battery for identifying the earliest changes in cognition. A systematic review found no effect of sex on the relationship between cognitive function and MedDiet adherence, although noted that not all studies fully considered sex differences (Kelly et al., 2020). This remains a topic warranting further research.

This study benefited from exploring the research question both cross-sectionally and longitudinally and shows the value of including repeated measures of dietary data and cognitive function in longitudinal cohort studies. The EPAD cohort offered a unique opportunity to directly compare participants living within and outside of the Mediterranean, who otherwise met the same eligibility criteria and represented similar spectrums of risk for AD. Ongoing cohort studies that similarly span regions could consider the addition of dietary data collection to supplement our knowledge in this area. Finally the EPAD cohort was established with cognitive function as a key outcome measure, with cognitive tasks thoroughly considered prior to inclusion (Ritchie et al., 2017). This rigorously designed cognitive test battery designed specifically to detect the earliest stages of AD is a key strength of both the EPAD cohort and this study investigating cognitive outcomes.

We applied three scoring methods to the dietary data to replicate the analytical approach taken in the EPIC-Norfolk study (Shannon et al., 2019). The MEDAS score is one of the most commonly used scores in MedDiet research hence was identified as the primary exposure variable. The two continuous scores were included to provide greater sensitivity which may be important particularly in non-Mediterranean regions where achieving traditional MEDAS cut-offs for each food group may be difficult for participants. Both continuous measures were used as the Pyramid is more widely used, and the MEDAS Continuous has been developed using cohorts recruiting participants in the United Kingdom.

One limitation of our study is that the dietary data questionnaire was exclusively focused on components of the MedDiet. As such it is missing several food groups required to calculate other dietary scores as well as overall energy intake. One consideration that may explain the general lack of association between the diet scores and global cognition, is that any benefits of the MedDiet are attenuated by consumption of unhealthy foods. A study of over 5,000 participants in the Chicago Health and Aging Project found that the MedDiet only had a significant effect on cognitive decline with a low and not a high Western diet score, a high score being an indicator of an unhealthy food consumption pattern (Agarwal et al., 2021). The Western diet is associated with cognitive impairment as well as disruption to hippocampal function and is important in our considerations of brain health (Kanoski and Davidson, 2011). A high glycaemic intake, particularly when included in the diet as an afternoon snack, has been associated with increased dementia risk for *APOE-*e4 carriers (Gentreau et al., 2020). It may be that we have an unmeasured effect of the Western diet in our results. This may be particularly relevant in the non-Mediterranean regions where the Western diet may be more prevalent, but may also have resulted in missing commonly eaten healthy foods not captured within the questionnaire.

Another limitation of this study is the self-report nature of the questionnaire, introducing the risk of recall bias, with participants forgetting elements of their diet when asked to report a general overview. This is a well-recognised problem in the nutritional field and method of data collection must be balanced between reduction of bias (such as using food diaries) and time considerations (brief recall questionnaires). Future research including dietary metabolomics should be considered to provide a more comprehensive overview of nutritional intake (Siervo et al., 2021).

Future research should further explore the associations of the MedDiet within and outside of the biogeographical Mediterranean region to explore the results from our sensitivity analysis. Research should also explore sex differences in the effects of the MedDiet to inform our understanding of who may benefit the most from this dietary pattern. Finally, research should investigate the impact of the Western diet on any effects of the MedDiet.

## 5 Conclusion

Our study found that the adherence to the MedDiet was associated with better performance on a measure of allocentric processing, both cross sectionally and longitudinally. Sensitivity analysis suggested that those living within the true biogeographical borders of the Mediterranean may have more general cognitive benefit associated with MedDiet adherence, although future research is needed. Understanding more about the associations between the MedDiet and brain health, particularly when and for how long adhering to the dietary pattern is important, will be critical both for designing public health strategies and providing individual patient advice.

## Data Availability

The original contributions presented in the study are included in the article/Supplementary Material, further inquiries can be directed to the corresponding author.

## References

[B1] Agarwal P. Dhana K. Barnes L. L. Holland T. M. Zhang Y. Evans D. A. (2021). Unhealthy foods may attenuate the beneficial relation of a Mediterranean diet to cognitive decline. Alzheimers Dement. 17, 1157–1165. 10.1002/alz.12277 33410584 PMC9764420

[B2] Berr C. Portet F. Carriere I. Akbaraly T. N. Feart C. Gourlet V. (2009). Olive oil and cognition: Results from the three-city study. Dement. Geriatr. Cogn. Disord. 28, 357–364. 10.1159/000253483 19887798 PMC2796327

[B3] Buysse D. J. Reynolds C. F. Monk T. H. Berman S. R. Kupfer D. J. (1989). The Pittsburgh sleep quality index: A new instrument for psychiatric practice and research. Psychiatry Res. 28, 193–213. 10.1016/0165-1781(89)90047-4 2748771

[B4] Chan D. Gallaher L. M. Moodley K. Minati L. Burgess N. Hartley T. (2016). The 4 mountains test: A short test of spatial memory with high sensitivity for the diagnosis of pre-dementia Alzheimer's disease. J. Vis. Exp., 116 54454. 10.3791/54454 PMC5092189 27768046

[B5] Chen K. H. M. Chuah L. Y. M. Sim S. K. Y. Chee M. W. L. (2010). Hippocampal region-specific contributions to memory performance in normal elderly. Brain Cogn. 72, 400–407. 10.1016/j.bandc.2009.11.007 20044193

[B6] Chen X. Liu Z. Sachdev P. S. Kochan N. A. O'Leary F. Brodaty H. (2022). Association of dietary patterns with cognitive function and cognitive decline in sydney memory and ageing study: A longitudinal analysis. J. Acad. Nutr. Diet. 122, 949–960.e15. 10.1016/j.jand.2021.10.018 34688967

[B7] Coelho-Júnior H. J. Trichopoulou A. Panza F. (2021). Cross-sectional and longitudinal associations between adherence to mediterranean diet with physical performance and cognitive function in older adults: A systematic review and meta-analysis. Ageing Res. Rev. 70, 101395. 10.1016/j.arr.2021.101395 34153553

[B8] De T. M. A. Fàbregues S. Bach-Faig A. Fornieles-Deu A. Medina F. X. Aguilar-Martínez A. (2021). Family meals, conviviality, and the mediterranean diet among families with adolescents. Int. J. Environ. Res. Public Health 18, 2499. 10.3390/ijerph18052499 33802507 PMC7967627

[B9] Estruch R. Ros E. Salas-Salvadó J. Covas M.-I. Corella D. Arós F. (2018). Primary prevention of cardiovascular disease with a mediterranean diet supplemented with extra-virgin olive oil or nuts. N. Engl. J. Med. 378, e34. 10.1056/NEJMoa1800389 29897866

[B10] EUROPEAN COMMISSION Natura 2000- Mediterranean biogeographical region. [Online]. Available https://ec.europa.eu/environment/nature/natura2000/biogeog_regions/maps/mediterranean.pdf . (Accessed August 21, 2021).

[B11] Féart C. Samieri C. Rondeau V. Amieva H. Portet F. Dartigues J.-F. (2009). Adherence to a Mediterranean diet, cognitive decline, and risk of dementia. JAMA 302, 638–648. 10.1001/jama.2009.1146 19671905 PMC2850376

[B12] Garcia-Closas R. Berenguer A. González C. A. (2007). Changes in food supply in Mediterranean countries from 1961 to 2001. Public Health Nutr. 9, 53–60. 10.1079/phn2005757 16480534

[B13] Gentreau M. Chuy V. Féart C. Samieri C. Ritchie K. Raymond M. (2020). Refined carbohydrate-rich diet is associated with long-term risk of dementia and Alzheimer's disease in apolipoprotein E ε4 allele carriers. Alzheimers Dement. 16, 1043–1053. 10.1002/alz.12114 32506713

[B14] Gutierrez L. Folch A. Rojas M. Cantero J. L. Atienza M. Folch J. (2021). Effects of nutrition on cognitive function in adults with or without cognitive impairment: A systematic review of randomized controlled clinical trials. Nutrients 13, 3728. 10.3390/nu13113728 34835984 PMC8621754

[B15] Hoscheidt S. Sanderlin A. H. Baker L. D. Jung Y. Lockhart S. Kellar D. (2021). Mediterranean and western diet effects on Alzheimer's disease biomarkers, cerebral perfusion, and cognition in mid-life: A randomized trial. Alzheimers Dement. 18, 457–468. 10.1002/alz.12421 34310044 PMC9207984

[B16] Hossain S. Beydoun M. A. Weiss J. Kuczmarski M. F. Evans M. K. Zonderman A. B. (2020). Longitudinal associations between dietary quality and Alzheimer's disease genetic risk on cognitive performance among African American adults. Br. J. Nutr. 124, 1264–1276. 10.1017/S0007114520001269 32248879 PMC7541564

[B17] Kanoski S. E. Davidson T. L. (2011). Western diet consumption and cognitive impairment: Links to hippocampal dysfunction and obesity. Physiol. Behav. 103, 59–68. 10.1016/j.physbeh.2010.12.003 21167850 PMC3056912

[B18] Karantzoulis S. Novitski J. Gold M. Randolph C. (2013). The repeatable battery for the assessment of neuropsychological Status (RBANS): Utility in detection and characterization of mild cognitive impairment due to Alzheimer's disease. Arch. Clin. Neuropsychol. 28, 837–844. 10.1093/arclin/act057 23867976

[B19] Kelly M. E. Loughrey D. G. Power J. M. Mcevoy C. Sheerin C. Pennie B. (2020). The impact of adherence to the traditional mediterranean diet and sex differences on global cognitive functioning: A systematic review and meta-analysis. J. Cogn. Enhanc. 4, 179–191. 10.1007/s41465-019-00143-6

[B20] Khalatbary A. R. (2013). Olive oil phenols and neuroprotection. Nutr. Neurosci. 16, 243–249. 10.1179/1476830513Y.0000000052 23406576

[B21] Khan U. A. Liu L. Provenzano F. A. Berman D. E. Profaci C. P. Sloan R. (2014). Molecular drivers and cortical spread of lateral entorhinal cortex dysfunction in preclinical Alzheimer's disease. Nat. Neurosci. 17, 304–311. 10.1038/nn.3606 24362760 PMC4044925

[B22] Knight A. Bryan J. Wilson C. Hodgson J. M. Davis C. R. Murphy K. J. (2016). The mediterranean diet and cognitive function among healthy older adults in a 6-month randomised controlled trial: The MedLey study. Nutrients 8, 579. 10.3390/nu8090579 27657119 PMC5037563

[B23] Martínez-González M. Hershey M. S. Zazpe I. Trichopoulou A. (2017). Transferability of the mediterranean diet to non-mediterranean countries. What is and what is not the mediterranean diet. Nutrients 9, 1226. 10.3390/nu9111226 29117146 PMC5707698

[B24] Martínez-Lapiscina E. H. Clavero P. Toledo E. Estruch R. Salas-Salvadó J. San Julián B. (2013). Mediterranean diet improves cognition: The PREDIMED-NAVARRA randomised trial. J. Neurol. Neurosurg. Psychiatry 84, 1318–1325. 10.1136/jnnp-2012-304792 23670794

[B25] Millman J. F. Okamoto S. Teruya T. Uema T. Ikematsu S. Shimabukuro M. (2021). Extra-virgin olive oil and the gut-brain axis: Influence on gut microbiota, mucosal immunity, and cardiometabolic and cognitive health. Nutr. Rev. 79, 1362–1374. 10.1093/nutrit/nuaa148 33576418 PMC8581649

[B26] Moodley K. Minati L. Contarino V. Prioni S. Wood R. Cooper R. (2015). Diagnostic differentiation of mild cognitive impairment due to Alzheimer's disease using a hippocampus-dependent test of spatial memory. Hippocampus 25, 939–951. 10.1002/hipo.22417 25605659

[B27] Moustafa B. Trifan G. Isasi C. R. Lipton R. B. Sotres-Alvarez D. Cai J. (2022). Association of mediterranean diet with cognitive decline among diverse hispanic or latino adults from the hispanic community health study/study of latinos. JAMA Netw. Open 5, e2221982. 10.1001/jamanetworkopen.2022.21982 35834250 PMC9284337

[B28] Nestor P. J. Fryer T. D. Ikeda M. Hodges J. R. (2003). Retrosplenial cortex (BA 29/30) hypometabolism in mild cognitive impairment (prodromal Alzheimer's disease). Eur. J. Neurosci. 18, 2663–2667. 10.1046/j.1460-9568.2003.02999.x 14622168

[B29] Panagiotakos D. B. Pitsavos C. Arvaniti F. Stefanadis C. (2007). Adherence to the Mediterranean food pattern predicts the prevalence of hypertension, hypercholesterolemia, diabetes and obesity, among healthy adults; the accuracy of the MedDietScore. Prev. Med. 44, 335–340. 10.1016/j.ypmed.2006.12.009 17350085

[B30] Radd-Vagenas S. Duffy S. L. Naismith S. L. Brew B. J. Flood V. M. Fiatarone Singh M. A. (2018). Effect of the mediterranean diet on cognition and brain morphology and function: A systematic review of randomized controlled trials. Am. J. Clin. Nutr. 107, 389–404. 10.1093/ajcn/nqx070 29566197

[B31] Richard E. Moll Van Charante E. P. Hoevenaar-Blom M. P. Coley N. Barbera M. Van G. A. (2019). Healthy ageing through internet counselling in the elderly (HATICE): A multinational, randomised controlled trial. Lancet. Digit. Health 1, e424–e434. 10.1016/S2589-7500(19)30153-0 33323224

[B32] Ritchie C. W. Molinuevo J. L. Truyen L. Satlin A. Van G. S. Lovestone S. (2016). Development of interventions for the secondary prevention of Alzheimer's dementia: The European prevention of Alzheimer's dementia (EPAD) project. Lancet. Psychiatry 3, 179–186. 10.1016/S2215-0366(15)00454-X 26683239

[B33] Ritchie C. W. Muniz-Terrera G. Kivipelto M. Solomon A. Tom B. Molinuevo J. L. (2020). The European prevention of Alzheimer’s dementia (EPAD) longitudinal cohort study: Baseline data release V500.0. J. Prev. Alzheimers Dis. 7, 8–13. 10.14283/jpad.2019.46 32010920

[B34] Ritchie K. Carrière I. Howett D. Su L. Hornberger M. O’Brien J. T. (2018). Allocentric and egocentric spatial processing in middle-aged adults at high risk of late-onset Alzheimer’s disease: The PREVENT dementia study. J. Alzheimers Dis. 65, 885–896. 10.3233/JAD-180432 30103333

[B35] Ritchie K. Ropacki M. Albala B. Harrison J. Kaye J. Kramer J. (2017). Recommended cognitive outcomes in preclinical Alzheimer's disease: Consensus statement from the European Prevention of Alzheimer's Dementia project. Alzheimers Dement. 13, 186–195. 10.1016/j.jalz.2016.07.154 27702619

[B36] Rowe C. C. Ng S. Ackermann U. Gong S. J. Pike K. Savage G. (2007). Imaging β-amyloid burden in aging and dementia. Neurology 68, 1718–1725. 10.1212/01.wnl.0000261919.22630.ea 17502554

[B37] Sacn (2018). SACN statement on diet, cognitive impairment and dementia. London, UK: SACN.

[B38] Samieri C. Grodstein F. Rosner B. A. Kang J. H. Cook N. R. Manson J. E. (2013). Mediterranean diet and cognitive function in older age. Epidemiol. Camb. Mass.) 24, 490–499. 10.1097/EDE.0b013e318294a065 PMC3674216 23676264

[B39] Scarmeas N. Anastasiou C. A. Yannakoulia M. (2018). Nutrition and prevention of cognitive impairment. Lancet. Neurol. 17, 1006–1015. 10.1016/S1474-4422(18)30338-7 30244829

[B40] Shannon O. M. Stephan B. C. M. Granic A. Lentjes M. Hayat S. Mulligan A. (2019). Mediterranean diet adherence and cognitive function in older UK adults: The European prospective investigation into cancer and nutrition-norfolk (EPIC-Norfolk) study. Am. J. Clin. Nutr. 110, 938–948. 10.1093/ajcn/nqz114 31204785

[B41] Siervo M. Shannon O. M. Llewellyn D. J. Stephan B. C. Fontana L. (2021). Mediterranean diet and cognitive function: From methodology to mechanisms of action. Free Radic. Biol. Med. 176, 105–117. 10.1016/j.freeradbiomed.2021.09.018 34562607

[B42] Solomon A. Kivipelto M. Molinuevo J. L. Tom B. Ritchie C. W. (2018). European prevention of Alzheimer’s dementia longitudinal cohort study (EPAD LCS): Study protocol. BMJ Open 8, e021017. 10.1136/bmjopen-2017-021017 PMC6318591 30782589

[B43] Tanaka T. Talegawkar S. A. Jin Y. Colpo M. Ferrucci L. Bandinelli S. (2018). Adherence to a mediterranean diet protects from cognitive decline in the invecchiare in chianti study of aging. Nutrients 10, 2007. 10.3390/nu10122007 30572567 PMC6316104

[B44] Tangney C. C. Kwasny M. J. Li H. Wilson R. S. Evans D. A. Morris M. C. (2011). Adherence to a Mediterranean-type dietary pattern and cognitive decline in a community population. Am. J. Clin. Nutr. 93, 601–607. 10.3945/ajcn.110.007369 21177796 PMC3041601

[B45] Tong T. Y. Wareham N. J. Khaw K. T. Imamura F. Forouhi N. G. (2016). Prospective association of the mediterranean diet with cardiovascular disease incidence and mortality and its population impact in a non-mediterranean population: The EPIC-norfolk study. BMC Med. 14, 135. 10.1186/s12916-016-0677-4 27679997 PMC5041408

[B46] Trichopoulou A. Kyrozis A. Rossi M. Katsoulis M. Trichopoulos D. La Vecchia C. (2015). Mediterranean diet and cognitive decline over time in an elderly Mediterranean population. Eur. J. Nutr. 54, 1311–1321. 10.1007/s00394-014-0811-z 25482573

[B47] Valls-Pedret C. Sala-Vila A. Serra-Mir M. Corella D. De T. R. Martínez-González M. Á. (2015). Mediterranean diet and age-related cognitive decline: A randomized clinical trial. JAMA Intern. Med. 175, 1094–1103. 10.1001/jamainternmed.2015.1668 25961184

[B48] Vareiro D. Bach-Faig A. Raidó Quintana B. Bertomeu I. Buckland G. Vaz De Almeida M. D. (2009). Availability of mediterranean and non-mediterranean foods during the last four decades: Comparison of several geographical areas. Public Health Nutr. 12, 1667–1675. 10.1017/S136898000999053X 19689838

[B49] Vilarnau C. Stracker D. M. Funtikov A. Da Silva R. Estruch R. Bach-Faig A. (2019). Worldwide adherence to mediterranean diet between 1960 and 2011. Eur. J. Clin. Nutr. 72, 83–91. 10.1038/s41430-018-0313-9 30487566

[B50] Wade A. T. Elias M. F. Murphy K. J. (2021). Adherence to a mediterranean diet is associated with cognitive function in an older non-mediterranean sample: Findings from the Maine-syracuse longitudinal study. Nutr. Neurosci. 24, 542–553. 10.1080/1028415X.2019.1655201 31432770

[B51] Yusufov M. Weyandt L. L. Piryatinsky I. (2017). Alzheimer's disease and diet: A systematic review. Int. J. Neurosci. 127, 161–175. 10.3109/00207454.2016.1155572 26887612

[B52] Zhang H. Hardie L. Cade J. (2021). Foods, nutrient intakes and mediterranean dietary pattern in midlife are not associated with reaction times: A longitudinal analysis of the UK Women’s cohort study. Br. J. Nutr. 125, 194–202. 10.1017/S0007114520002287 32594947

