# Supplementary material for: Mediterranean diet score is associated with greater allocentric processing in the EPAD LCS cohort: A comparative analysis by biogeographical region
Source: Front Aging. 2022 Dec 9;3:1012598. doi: 10.3389/fragi.2022.1012598 (PMC9780498; doi:10.3389/fragi.2022.1012598)
Supplement: Supplementary file 1 [file DataSheet1.docx]

Supplementary Material
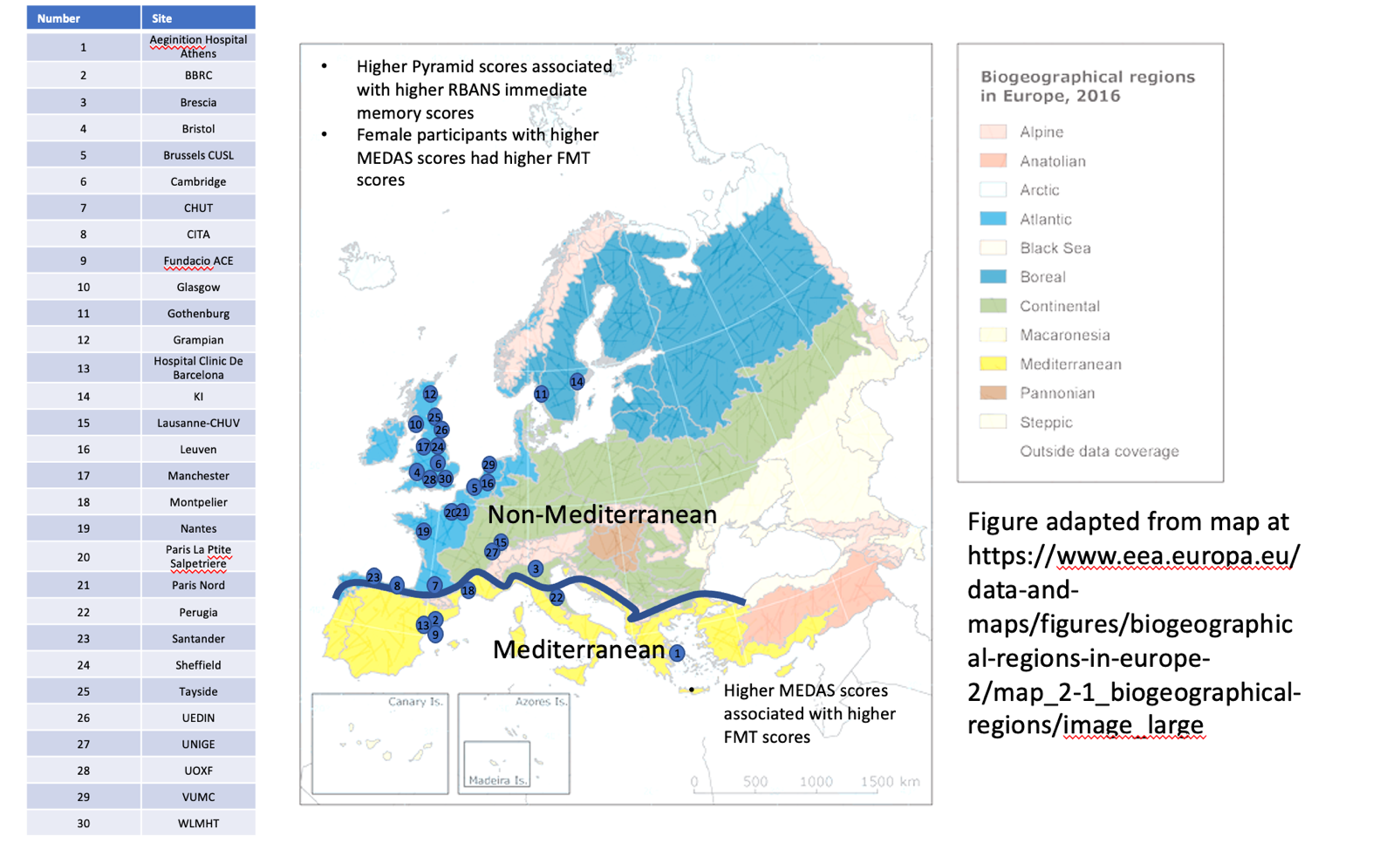


Supplementary Figure 1. Figure depicting site location by biogeographical regions in Europe alongside summary of results within and outside of the biogeographical European Mediterranean region.

1. European Commission. *Natura 2000- Mediterranean biogeographical region* [cited 2022 19th August 2021]; Available from: <https://ec.europa.eu/environment/nature/natura2000/biogeog_regions/maps/mediterranean.pdf>.

| **Site Name** | **City** | **Country** | **Mediterranean** | **Number of participants** |
| --- | --- | --- | --- | --- |
| Aeginition Hospital Athens | Athens | Greece | Yes | 27 |
| BBRC | Barcelona | Spain | Yes | 253 |
| Brescia | Brescia | Italy | No | 45 |
| Bristol | Bristol | UK | No | 48 |
| Brussels CUSL | Brussels | Belgium | No | 6 |
| Cambridge | Cambridge | UK | No | 5 |
| CHUT | Toulouse | France | Borderline | 215 |
| CITA | San Sebastian | Spain | No | 303 |
| Fundacio ACE | Barcelona | Spain | Yes | 84 |
| Glasgow | Glasgow | UK | No | 49 |
| Gothenburg | Gothenburg | Sweden | No | 9 |
| Grampian | Aberdeen | UK | No | 49 |
| Hospital Clinic De Barcelona | Barcelona | Spain | Yes | 6 |
| KI | Stockholm | Sweden | No | 41 |
| Lausanne-CHUV | Lausanne | Switzerland | No | 2 |
| Leuven | Leuven | Belgium | No | 30 |
| Manchester | Manchester | UK | No | 7 |
| Montpelier | Montpelier | France | Yes | 74 |
| Nantes | Nantes | France | No | 22 |
| Paris La Ptite Salpetriere | Paris | France | No | 70 |
| Paris Nord | Paris | France | No | 56 |
| Perugia | Perugia | Italy | Borderline | 14 |
| Santander | Santander | Spain | No | 4 |
| Sheffield | Sheffield | UK | No | 10 |
| Tayside | Dundee | UK | No | 103 |
| UEDIN | Edinburgh | UK | No | 104 |
| UNIGE | Geneva | Switzerland | No | 119 |
| UOXF | Oxford | UK | No | 76 |
| VUMC | Amsterdam | The Netherlands | No | 223 |
| WLMHT | London | UK | No | 38 |
| **Supplementary Table S1:** Classification of site into Mediterranean, non-Mediterranean and borderline according to the EU commission [1]. Borderline countries were included as Mediterranean countries after consensus discussion by the study authors. A sensitivity analysis was run excluding these two sites from the analysis. | | | | |

| **Food component** | **MEDAS Rule** | **MEDAS** | **MEDAS-C Rule** | **MEDAS Continuous^±^** | **Pyramid Rule** | **Pyramid** |
| --- | --- | --- | --- | --- | --- | --- |
| Olive oil (consumption) | Rule 1 | 1 point: Consumption  0 points: Non-consumption | Rule 1 | 1 point: Consumption  0 points: Non-consumption | Rule 15 | 1 point: Consumption  0 points: Non-consumption |
| Olive oil (volume)^¶^ | Rule 2 | 1 point: ≥4 Tbsp/d  0 points: <4 Tbsp/d | Rule 2 | 1 point: ≥4 Tbsp/d  0 points: <4 Tbsp/d | NA | |
| Vegetables | Rule 3 | 1 point: ≥2 portions/d  0 points: <2 portions/d | Rule 3 | 1 point: ≥2 portions/d  0 points: 0 portions/d | Rule 1 | 1 point: ≥6 portions/d  0 points: 0 portions/d |
| Fruit | Rule 4 | 1 point: ≥3 portions/d  0 points: <3 portions/d | Rule 4 | 1 point: ≥3 portions/d  0 points: 0 portions/d | Rule 3 | 1 point: 3-6portions/d  0 points: 0 portions/d  0.5 points: overconsumption^#^ |
| Red meat | Rule 5 | 1 point: <1 portion/d  0 points: ≥1 portion/d | Rule 5 | 1 point: <1 portion/d  0 points: ≥2 portions/d | Rule 8 | 1 point: <2 portion/wk  0 points: ≥2 portions/wk |
| Dairy | Rule 6 | 1 point: <1 portion/d  0 points: ≥1 portion/d | Rule 6 | 1 point: <1 portion/d  0 points: ≥2 portions/d | Rule 6 | 1 point: 1.2-2.5 portions/d  0 point: 0 portions  0.5: overconsumption^#^ |
| Carbonated/sweet drinks | Rule 7 | 1 point: <1 portion/d  0 points: ≥1 portion/d | Rule 7 | 1 point: <1 portion/d  0 points: ≥2 portions/d | NA | |
| Alcohol (wine only for MEDAS, MEDAS continuous) | Rule 8 | 1 point: ≥7 portions/wk  0 points: <7 portions /wk | Rule 8 | 1 point: ≥7 portions/wk  0 points: 0 portions /wk | Rule 14 | 1 point: 1.5-2.5 portions/d (male)  1 point: 0.5-1.5 portions/d (female)  0 points: 0 portions/d  0.5: overconsumption^#^ |
| Pulses | Rule 9 | 1 point: ≥3 portions/wk  0 points: <3 portions/wk | Rule 9 | 1 point: ≥3 portions/wk  0 points: 0 portions/wk | Rule 2 | 1 point: ≥2 portions/wk  0 points: 0 portions/wk |
| Fish/seafood | Rule 10 | 1 point: ≥3 portions/wk  0 points: <3 portions/wk | Rule 10 | 1 point: ≥3 portions/wk  0 points: 0 portions/wk | Rule 7 | 1 point: ≥2 portions/wk  0 points: 0 portions/wk |
| Pastries | Rule 11 | 1 point: <2 portions/wk  0 points: ≥2 portions/wk | Rule 11 | 1 point: <2 portion/wk  0 points: ≥4 portions/wk | NA | |
| Nuts | Rule 12 | 1 point: ≥3 portions/wk  0 points: <3 portions/wk | Rule 12 | 1 point: ≥3 portions/wk  0 points: 0 portions/wk | Rule 4 | 1 point: 1-2 portions/d  0 points: 0 portions/d  0.5 points: overconsumption^#^ |
| White meat | Rule 13 | 1 point: more white meat  0 points: more red meat | Rule 13 | 1 point: more white meat  0 points: more red meat | Rule 10 | 1 point: 1.5-2.5 portions/wk  0 point: 0 portions/wk  0.5: overconsumption^#^ |
| Sofrito | Rule 14 | 1 point: ≥2 portions/wk  0 points: <2 portions/wk | Rule 14 | 1 point: ≥2 portions/wk  0 points: 0 portions/wk | NA | |
| Cereals | NA | | | | Rule 5 | 1 point: 3-6 portions/d  0 points: 0 portions/d  0.5 points: overconsumption^#^ |
| Processed meat | NA | | | | Rule 9 | 1 point: <2 portion/wk  0 points: ≥2 portions/wk |
| Potato | NA | | | | Rule 12 | 1 point: ≤3 portions/wk  0 points: >3 portions/wk |
| Sweets | NA | | | | Rule 13 | 1 point: ≤2 portions/wk  0 points: >2 portions/wk |
| **Supplementary Table S2:** Scoring criteria for MedDiet scores (MEDAS, MEDAS Continuous and Pyramid scores. ^¶^Data collected as binary so continuous scoring could not be applied for MEDAS continuous or Pyramid scores. ^±^Scores assigned on continuous scale between 0 and 1 with servings required for 0 and 1 indicated in table. ^#^Two-fold higher than mid-point of recommended intake. Note egg is usually included in the calculation of the Pyramid score, however there is no data on egg consumption collected in the EPAD LCS and this component was not included in the Pyramid score calculation (Rule 11) Abbreviations: Tbsp: Tablespoon; d: day; wk: week | | | | | | |

| Cognitive Test | **Male Mediterranean** | | **Male Non-Mediterranean** | | **Female Mediterranean** | | **Female Non-Mediterranean** | |
| --- | --- | --- | --- | --- | --- | --- | --- | --- |
|  | Unadjusted | Fully Adjusted | Unadjusted | Fully Adjusted | Unadjusted | Fully Adjusted | Unadjusted | Fully Adjusted |
| 4 Mountains Test | ***β: 0.24***  ***SE: 0.11***  ***p: 0.03**** | β: 0.08  SE: 0.10  p: 0.45 | β: 0.07  SE: 0.08  p: 0.39 | β: -0.02  SE: 0.08  p: 0.78 | β: 0.18  SE: 0.10  p: 0.09 | β: 0.19  SE: 0.10  p: 0.05 | ***β: 0.22***  ***SE: 0.07***  ***p: 0.003***** | ***β: 0.22***  ***SE: 0.07***  ***p: 0.003***** |
| RBANS Total Scale | β: -0.11  SE: 0.59  p: 0.86 | β: -0.66  SE: 0.59  p: 0.26 | β: 0.19  SE: 0.36  p: 0.60 | β: -0.17  SE: 0.36  p: 0.63 | β: 0.24  SE: 0.54  p: 0.67 | β: 0.36  SE: 0.49  p: 0.46 | β: 0.35  SE: 0.34  p: 0.30 | β: -0.08  SE: 0.33  p: 0.79 |
| RBANS Attention Index | β: 0.72  SE: 0.65  p: 0.26 | β: 0.16  SE: 0.65  p: 0.81 | β: 0.15  SE: 0.42  p: 0.72 | β: -0.08  SE: 0.44  p: 0.85 | β: 0.20  SE: 0.59  p: 0.73 | β: 0.27  SE: 0.56  p: 0.63 | β: -0.03  SE: 0.38  p: 0.94 | β: -0.41  SE: 0.38  p: 0.29 |
| RBANS Delayed Memory Index | β: -0.29  SE: 0.65  p: 0.66 | β: -0.82  SE: 0.67  p: 0.22 | β: 0.23  SE: 0.43  p: 0.59 | β: -0.15  SE: 0.42  p: 0.73 | β: 0.63  SE: 0.64  p: 0.33 | β: 0.77  SE: 0.62  p: 0.21 | β: 0.31  SE: 0.34  p: 0.36 | β: 0.09  SE: 0.34  p: 0.80 |
| RBANS Immediate Memory Index | β: -0.25  SE: 0.64  p: 0.70 | β: -0.68  SE: 0.64  p: 0.29 | β: 0.73  SE: 0.39  p: 0.06 | β: 0.23  SE: 0.38  p: 0.55 | β: 0.43  SE: 0.54  p: 0.42 | β: 0.41  SE: 0.53  p: 0.44 | β: 0.59  SE: 0.33  p: 0.07 | β: 0.22  SE: 0.32  p: 0.50 |
| RBANS Language Index | β: -0.06  SE: 0.34  p: 0.85 | β: -0.24  SE: 0.36  p: 0.51 | β: -0.30  SE: 0.27  p: 0.27 | β: -0.34  SE: 0.28  p: 0.22 | β: -0.05  SE: 0.38  p: 0.89 | β: 0.19  SE: 0.38  p: 0.62 | β: 0.05  SE: 0.28  p: 0.86 | β: -0.11  SE: 0.28  p: 0.69 |
| RBANS Visuo-constructional Index | β: -0.09  SE: 0.64  p: 0.90 | β: -0.50  SE: 0.67  p: 0.45 | β: 0.01  SE: 0.40  p: 0.98 | β: -0.18  SE: 0.40  p: 0.65 | β: -0.07  SE: 0.60  p: 0.91 | β: -0.02  SE: 0.58  p: 0.97 | β: 0.18  SE: 0.36  p: 0.61 | β: -0.16  SE: 0.35  p: 0.64 |
| **Supplementary Table S3:** Linear regression models of MEDAS score with cognitive outcomes living in male and female participants by region. Unadjusted Model: MEDAS Score; Fully Adjusted Model: MEDAS Score, age, education, family history, APOE, CAIDE, smoking, sleep, BMI, hypertension, hypercholesterolemia, hyperglycaemia, diabetes, stroke, antihypertensive medication use, diabetic medication use. *p<0.05; **p<0.01; ***p<0.001 | | | | | | | | |

| Cognitive Test | **Mediterranean** | | | | | |
| --- | --- | --- | --- | --- | --- | --- |
|  | **MEDAS** | | **MEDAS Continuous** | | **Pyramid** | |
|  | Unadjusted | Fully Adjusted | Unadjusted | Fully Adjusted | Unadjusted | Fully Adjusted |
| 4 Mountains Test | ***β: 0.32***  ***SE: 0.10***  ***p: 0.001***** | ***β: 0.24***  ***SE: 0.09***  ***p: 0.008***** | β: 0.07  SE: 0.12  p: 0.58 | β: -0.03  SE: 0.11  p: 0.77 | β: 0.05  SE: 0.12  p: 0.65 | β: 0.06  SE: 0.11  p: 0.61 |
| RBANS Total Scale | ***β: 1.74***  ***SE: 0.47***  ***p: 0.0002****** | ***β: 1.16***  ***SE: 0.44***  ***p: 0.009***** | ***β: 2.02***  ***SE: 0.56***  ***p: 0.0004****** | ***β: 1.49***  ***SE: 0.52***  ***p: 0.005***** | β: 0.94  SE: 0.58  p: 0.11 | β: 0.80  SE: 0.54  p: 0.14 |
| RBANS Attention Index | ***β: 1.81***  ***SE: 0.57***  ***p: 0.002***** | ***β: 1.22***  ***SE: 0.56***  ***p: 0.03**** | ***β: 1.86***  ***SE: 0.69***  ***p: 0.007***** | ***β: 1.35***  ***SE: 0.66***  ***p: 0.04**** | β: 0.28  SE: 0.71  p: 0.70 | β: -0.08  SE: 0.68  p: 0.90 |
| RBANS Delayed Memory Index | ***β: 1.51***  ***SE: 0.57***  ***p: 0.008***** | β: 0.85  SE: 0.56  p: 0.13 | ***β: 1.76***  ***SE: 0.68***  ***p: 0.01**** | β: 1.22  SE: 0.66  p: 0.06 | β: 0.88  SE: 0.70  p: 0.21 | β: 0.85  SE: 0.68  p: 0.21 |
| RBANS Immediate Memory Index | β: 0.98  SE: 0.51  p: 0.06 | β: 0.53  SE: 0.49  p: 0.29 | β: 1.14  SE: 0.61  p: 0.06 | β: 0.79  SE: 0.58  p: 0.18 | β: 0.61  SE: 0.63  p: 0.33 | β: 0.68  SE: 0.60  p: 0.26 |
| RBANS Language Index | ***β: 0.85***  ***SE: 0.32***  ***p: 0.009***** | ***β: 0.68***  ***SE: 0.33***  ***p: 0.04**** | ***β: 1.39***  ***SE: 0.38***  ***p: 0.0003****** | ***β: 1.30***  ***SE: 0.38***  ***p: 0.0008****** | β: 0.34  SE: 0.40  p: 0.39 | β: 0.44  SE: 0.40  p: 0.28 |
| RBANS Visuo-constructional Index | ***β: 1.53***  ***SE: 0.54***  ***p: 0.005***** | ***β: 1.20***  ***SE: 0.54***  ***p: 0.03**** | ***β: 1.51***  ***SE: 0.65***  ***p: 0.02**** | β: 0.89  SE: 0.65  p: 0.17 | β: 1.24  SE: 0.66  p: 0.06 | β: 0.96  SE: 0.67  p: 0.15 |
| **Supplementary Table S4:** Sensitivity analysis of linear regression models of MedDiet scores (MEDAS, MEDAS Continuous and Pyramid) with cognitive outcomes living in Mediterranean countries with Toulouse and Perugia excluded (FMT: n=262; RBANS n=393). Unadjusted Model: MedDiet Score; Fully Adjusted Model: MedDiet Score, sex, age, education, family history, APOE, physical activity, smoking, sleep, BMI, hypertension, hypercholesterolemia, hyperglycaemia, diabetes, stroke, antihypertensive medication use, diabetic medication use. *p<0.05; **p<0.01; ***p<0.001. | | | | | | |

| RBANS | | | | |
| --- | --- | --- | --- | --- |
| Visit | Baseline  (n= 1844) | Year 1  (n=1133) | Year 2  (n=386) | Year 3  (n=88) |
| MEDAS Score | 6.86 (1.66) | 6.81 (1.80) | 6.94 (1.57) | 6.70 (1.74) |
| RBANS total score | 102.00 (15.36) | 103.29 (13.80) | 107.53 (13.48) | 105.90 (12.52) |
| FMT | | | | |
| Visit | Baseline  (n= 1151) | Year 1  (n=564) | Year 2  (n=209) | Year 3  (n=15) |
| MEDAS Score | 6.82 (1.65) | 6.77 (1.84) | 6.87 (1.47) | 7.13 (1.55) |
| FMT total score | 9.23 (2.57) | 9.57 (2.61) | 10.12 (2.56) | 9.27 (3.22) |
| **Supplementary Table S5:** Summary of MEDAS scores, RBANS and FMT total scores (mean (SD)) by visit. | | | | |
